# Evolution of nest architecture in tyrant flycatchers and allies

**DOI:** 10.1098/rstb.2022.0148

**Published:** 2023-08-28

**Authors:** David Ocampo, Thilina N. De Silva, Catherine Sheard, Mary Caswell Stoddard

**Affiliations:** ^1^ Department of Ecology and Evolutionary Biology, Princeton University, Princeton, NJ 08544, USA; ^2^ Palaeobiology Research Group, University of Bristol, Bristol BS8 1TQ, UK

**Keywords:** Tyrannida, flycatcher, nest type, nest architecture, cup, dome

## Abstract

Innovations in nest design are thought to be one potential factor in the evolutionary success of passerine birds (order: Passeriformes), which colonized new ecological niches as they diversified in the Oligocene and Miocene. In particular, tyrant flycatchers and their allies (parvorder: Tyrannida) are an extremely diverse group of New World suboscine passerines occupying a wide range of habitats and exhibiting substantial extant variation in nest design. To explore the evolution of nest architecture in this clade, we first described nest traits across the Tyrannida phylogeny and estimated ancestral nest conditions. We then quantified macroevolutionary transition rates between nest types, examined a potential coevolutionary relationship between nest type and habitat, and used phylogenetic mixed models to determine possible ecological and environmental correlates of nest design. The Tyrannida ancestor probably built a cup nest in a closed habitat, and dome nests independently evolved at least 15 times within this group. Both cup- and dome-nesting species diversified into semi-open and open habitats, and we did not detect a coevolutionary relationship between nest type and habitat. Furthermore, nest type was not significantly correlated with several key ecological, life-history and environmental traits, suggesting that broad variation in Tyrannida nest architecture may not easily be explained by a single factor.

This article is part of the theme issue ‘The evolutionary ecology of nests: a cross-taxon approach’.

## 1. Introduction

Passeriformes (passerines, or ‘perching birds’) is the largest order of birds, comprising approximately 60% of extant avian species and occupying a wide range of ecological niches worldwide. This clade's ecological and evolutionary success has been attributed to many potential factors [1–3], including innovation in nest-building behaviours [4–10]. A well-built nest represents a key component of successful reproduction in many avian species, protecting eggs and chicks from predation and environmental pressures [11–14]. Unlike non-passerine lineages, which exhibit strong evolutionary conservatism in nest design [4,15], some passerine species have explored and occupied many different nest-building micro-niches (e.g. [9,16–18]), and traits related to nest construction seem to be highly labile within multiple subclades of this radiation [11,15,16,19,20].

Modern passerines appear to have evolved from cavity-nesting ancestors [21,22]. Cavity nesting can require substantial morphological or ecological specialization [23,24] and thus might limit a species' ecological tolerance, curtailing its ability to expand its range or to persist through environmental changes in habitat conditions [25,26]. To overcome these challenges, early modern passerines probably constructed dome nests outside of cavities [27,28], with several lineages subsequently acquiring the ability to reproduce in cup-shaped nests [9]. Dome nests (i.e. nests constructed with roofs) are thought to provide substantial protection from the environment and predators [16,29,30], but they also restrict breeding opportunities and potentially limit a species’ ecological niche [12,30]. Open cup nests, on the other hand, are considered easier to build than dome nests [23] and thus potentially facilitate the colonization of new niches [9]. The general drivers of variation in nest structure (i.e. cup versus dome), however, are relatively unknown, and evidence for widespread macroevolutionary consequences of innovations in nesting strategy is mixed [7,31].

**Figure 1.**
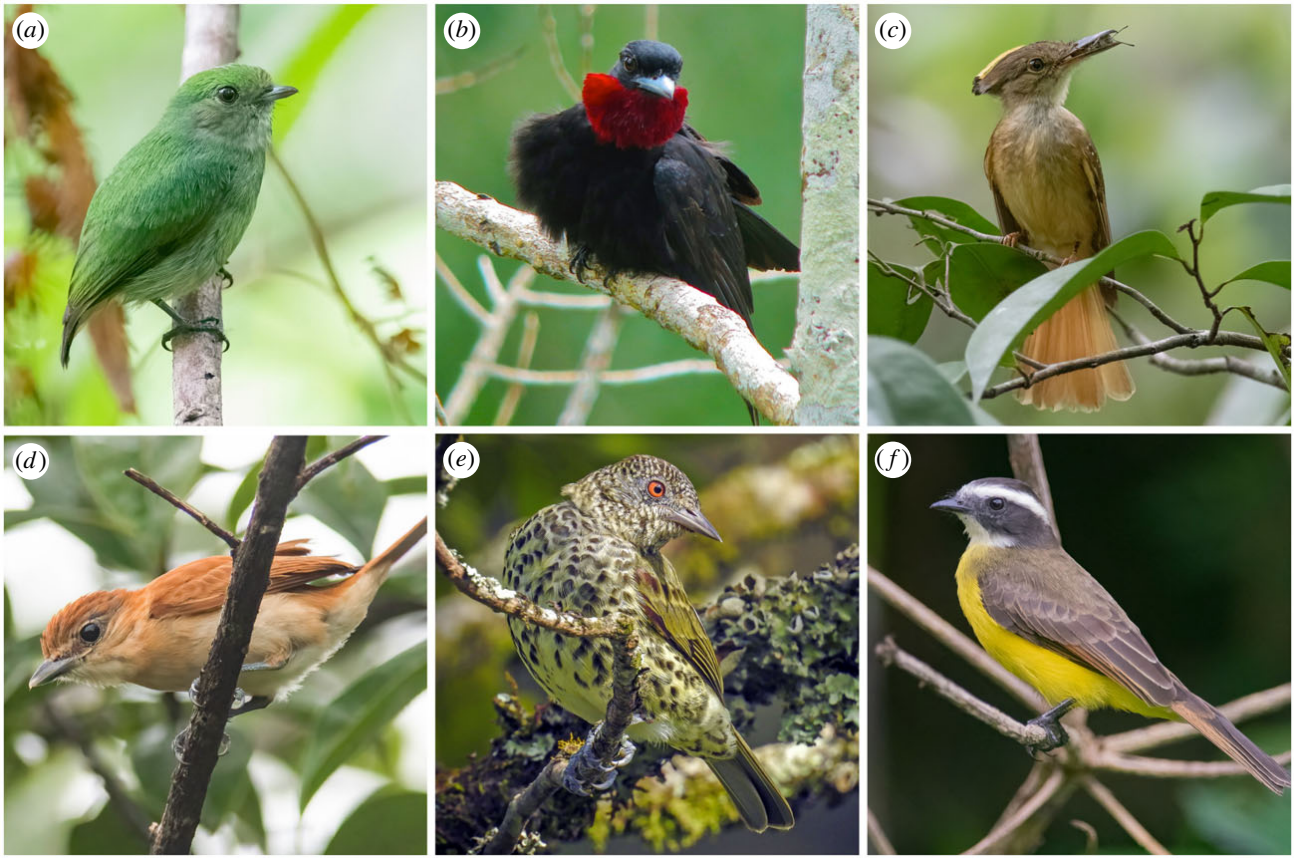
Bird species representing the different families in the parvorder Tyrannida. (*a*) Manakins–Pipridae (blue-capped manakin–*Lepidothrix coronata*), (*b*) cotingas–Cotingidae (purple-throated fruitcrow–*Querula purpurata*), (*c*) royal flycatchers–Onychorhynchidae (royal flycatcher–*Onychorhynchus coronatus*), (*d*) tityras–Tityridae (cinnamon becard–*Pachyramphus cinnamomeus*), (*e*) sharpbill–Oxyruncidae (sharpbill–*Oxyruncus cristatus*), and (*f*) tyrant flycatchers–Tyrannidae (rusty-margined flycatcher–*Myiozetetes cayanensis*). Photo credits: (*a-d*,*f*) Daniel Field, (*e*) Aisse Gaertner.

The suboscine passerines—and particularly the parvorder Tyrannida—provide a robust system in which to examine the evolutionary causes and consequences of innovations in nest architecture. The Tyrannida [32,33] are small Neotropical insectivores. They are found in a variety of different habitats and exhibit a range of breeding strategies, from polygyny and primarily female parental care to monogamy and shared biparental care [34]. The Tyrannida clade includes the most diverse avian family, the tyrant flycatchers (Tyrannidae) [31], as well as the manakins (Pipridae), cotingas (Cotingidae), royal flycatchers (Onychorhynchidae), tityras (Tityridae) and the sharpbill (*Oxyruncus cristatus*; family Oxyruncidae) (figure 1). Early Tyrannida birds inhabited interior forests in the Oligocene (*ca* 30 Ma), followed by subsequent divergence events in forest habitats and an explosive radiation that correlates in time with expansion into semi-open and open habitats in the mid-Miocene (*ca* 15 Ma), particularly in the tityras (Tityridae) and several lineages of the tyrant flycatchers (Tyrannidae) [35]. In addition to having unusually high inter-lineage variation in diversification rates [35–37], Tyrannida also contains many cup- and dome nesters, with closely related species sometimes exhibiting considerable nest type variation [34]. Thus, Tyrannida is a compelling group in which to perform a comparative analysis of nest type evolution: the substantial nest diversity within this group can be studied at a focused taxonomic scale, without the need to control for ecological factors that may vary widely across a broader taxonomic sample [38,39].

One of the main potential drivers of macroevolutionary shifts in avian nest architecture is nesting habitat (e.g. [4,5,22,39]). For example, birds nesting in open habitats are more exposed to environmental conditions such as solar radiation, wind and rain. On the other hand, birds nesting in closed habitats (i.e. dense forested vegetation) may be more protected from the elements; in addition, they have access to a greater range of nest locations and may be less exposed to predators compared to species nesting in open habitats or on the ground [26,40,41]. The influence of habitat on nest architecture, however, has rarely been tested at broader phylogenetic scales (for example, a parvorder); the effects of climate (e.g. [6,41]) or urbanization (e.g. [7]) are more commonly considered. With respect to climate, one study of Australian passerines [42] determined that dome nesting is more common in hot, dry regions with limited plant canopy cover. Beyond habitat, though, a number of additional ecological and life-history factors could influence nest design. These include other aspects of nest architecture (such as nest height and location) and a suite of ecological and life-history traits, including clutch size, adult body mass, flight behaviour, beak morphology and territorial behaviour (summarized in table 1). In addition, ecological interactions (like predation) and environmental factors (such as elevation, latitude, temperature, precipitation and range size; table 1) could affect nest architecture directly [6,10,63] if, for example, nest predation decreased with elevation [64] or if birds build dome nests to escape extreme cold [65] or heat [29]. In turn, a species' nest type might reflect its ability to tolerate or disperse in a wide range of environmental conditions [6]: cavity nesters may be more constrained since they may be more limited by nest-site availability [66], for example, than cup- or dome-nesting species [67]. An examination of the evolutionary link between habitat and nest type would therefore need to account for these co-varying ecological traits; it should also incorporate alternate measures of niche differentiation such as temperature and elevation that go beyond habitat type (see table 1 for a compiled summary of specific hypotheses and predictions).

**Table 1.** Trait definitions and summarized hypotheses about potential ecological, life-history and environmental correlates of nest type variation. (Note that nest location (five states) was not included in the phylogenetic mixed models exploring correlations with nest type variation; however, we included nest location in the ancestral state reconstruction illustrated in figure 1.)

	trait	definition of variables	hypotheses	key references
nest features	nest type	cup: nests that are rounded, with a central depression and no roof; dome: enclosed nests with a roof	shifts between the two basic nest types may be associated with habitat and a suite of other ecological, life-history and environmental traits	[14,16,21,43,44]
	nesting habitat	closed: dense habitats in forest; semi-open: forest edges, dense understory, thickets, or shrubland; open: deserts, grassland, low shrubs, rocky habitats, seashores, and cities	a suitable nest habitat involves an appropriate microclimate for embryo development and raising offspring, in a location that minimizes the risk of predation. Transitions to semi-open and open habitats should be more frequent in species with versatile cup nests that can be more easily hidden in the ground or in vegetation, to escape predators and harsh climate conditions. Closed habitats (forests) should offer a greater diversity of nest locations	[4,26,40]
	nest location	branch: nest partially supported on the vegetation; hanging: pensile in branches; ground: fully supported on the ground; banks and rocks: fully supported above ground in banks, trunks or rock surfaces; cavity: the nest structure rests inside a natural or artificial cavity	choosing a safe nesting site is important for reproductive success, and some birds have been shown to select their nest sites to reduce the risk of predation. The variety of vegetation in closed habitats should allow for a high diversity in nest locations, especially for cup nests that can be supported in or on different substrates	[4,14,15,45]
	nest height	average nest height (m) above the ground	ground-nesting birds select sites that minimize heat loss in cool environments and prevent overheating in warm environments, thereby creating an optimal microclimate in which to raise offspring. Cup nests are expected to be found at a variety of heights, particularly in closed habitats—where they can be located on the ground all the way up to the canopy. Nest height is sometimes considered to be a proxy for predation risk, with the greatest risk to ground-nesting birds—although whether this proxy is valid depends on many factors, including the suite of principal predators	[4,16,45,46]
ecological and life-history factors	clutch size	average clutch size	clutch size is one of the most important life-history traits and can be highly variable among birds. Cups (or open nests in general) may allow bigger clutch sizes compared with domes	[47–49]
	adult body mass	adult body mass (g)	small body mass in passerines allowed many species to invade and nest in almost every terrestrial habitat in the world. The problems of predation, environmental stress and energy balance are greatest for small birds such as passerines. In some passerines, enclosed (dome) nests are more likely to be built by smaller species, and larger species are more likely to build shallow nests supported on a surface	[6,11,29,44,50]
	flight ability	hand-wing index (HWI): 100*D_K_/*L*_w_, where *D*_K_ is Kipp's distance (the distance between the tip of the first secondary feather and the tip of the longest primary feather) and *L*_w_ is wing length [51]	in small birds, more elliptical wings (i.e. lower HWI) might provide more efficient manoeuvrability, which may be advantageous during nest building. For example, birds could access more obscure nesting locations (i.e. hanging, cavities) and invest in more complex structures (i.e. domes)	[4,52]
	beak dimensions	total beak length/beak depth, as a proportional proxy of a two-dimensional simplified beak size. Length and depth are two basic dimensions to describe long- or short-billed species [53]	the beak is directly linked with the mechanics of nest building. Specific associations between beak and nest type have rarely been reported, but dimensions of the beak could modulate nest complexity or nest material selection, influencing the structural features of nests. We hypothesize that domes are built principally by species with sharp beaks (i.e. higher beak dimensions)	[21,54,55]
	territoriality	none or weak or strong, based on studies searching for terms: territor∗, year-round territor∗, long-term territor∗, stable territor∗, breeding territor∗, flock territor∗, non-territor∗ [51]	nesting strategies can be associated with territorial behaviour when competition for nest sites is intense. This was tested in obligate secondary cavity-nesting species and two related species with more flexible nesting strategies in the same avian family. We hypothesize that dome nesters would be more territorial since competition for nest sites is likely to be more intense	[12,30,56].
environmental factors	elevation	average elevation (m)	predation and abiotic factors vary along elevational and latitudinal gradients. Birds build larger, less porous and more heavily insulated nests in colder environments associated with higher latitudes and altitudes. Nests that meet these criteria are often cup nests: cup nesters are likely to be predominant at higher elevation and latitudes	[14,57–60]
	latitude	centroid latitude (decimal degrees)		[46,61,62]
	temperature	average temperature (°C)	nest type might be associated with differences in niche width. Dome nests might offer superior protection against extreme climatic conditions. Alternatively, dome nests may be the ancestral architecture in passerines, adapted to very specific habitats. In that scenario, cup nests may have facilitated greater environmental tolerance because they can be flexibly built in a greater range of microhabitats	[4,6,7,14]
		annual variation (range) in temperature (°C)		
	precipitation	average precipitation (mm)		
		annual variation (range) in precipitation (mm)		
	range size	km^2^	shifts between dome and cup nests potentially allowed species to colonize new habitats in different environmental conditions. Thus, species that build a more versatile nest type may occupy a bigger range. Dome nesters have been shown to occupy smaller range sizes in Australian passerines	[6]

Here, we investigate the evolution of nest architecture and habitat in Tyrannida by first surveying nest structure and location across 466 species, a sample that represents 75% of currently described species and 95% of currently described genera. We then use Bayesian phylogenetic methods to estimate the ancestral nesting state of this clade, to quantify transition rates between nest architecture strategies, and to assess possible coevolutionary dynamics between nest architecture and habitat type. Finally, we use phylogenetic mixed models to determine whether nest type is correlated with diverse ecological, life-history and environmental traits.

## 2. Methods

### (a) Study system and data collection

#### (i) Study system

We studied a monophyletic lineage comprising tyrant flycatchers and allies in the suboscine parvorder Tyrannida [32,33]. We followed the *Handbook of the Birds of the World* and BirdLife International [68] for taxonomic descriptions and used Jetz *et al*. [69] for phylogenetic data. Tyrannida includes species that have been recently categorized into six families [70,71]: Pipridae, Cotingidae, Onychorhynchidae, Tityridae, Oxyruncidae and Tyrannidae, the last of which is the most speciose family of birds in the world [34]. Breeding strategies are mixed in Tyrannida: polygyny and primarily female parental care are very common in some families (Pipridae, Cotingidae), while monogamy and shared biparental care are typical in others (Onychorhynchidae, Tityridae, Oxyruncidae and Tyrannidae). Correspondingly, males and females may vary in their contributions to nest building, though there is a substantial lack of knowledge of nest-building behaviours for most of the species in the clade [34].

#### (ii) Nest design

We searched for nest architecture and nest location information for each species of Tyrannida. We principally used the website HBW Alive [72], supplemented with primary literature on nest descriptions to build our dataset S1 (electronic supplementary material, S2). After a detailed literature search, we were able to compile nest descriptions for 466 species (approx. 75%), encompassing 95% of the genera in this clade (table 2). We assigned each species in this dataset to one of two basic nest types: cup (i.e. cup-shaped nests that are rounded, with a central depression and no roof, *n* = 339 species) or dome (i.e. enclosed, constructed nests with a roof, *n* = 127 species). We then scored nest location as branch, hanging, ground, banks or rocks (i.e. fully supported off of the ground) or cavity, following the nest descriptions of neotropical birds given by Simon & Pacheco [73]. We categorized the nests of genus *Tityra* as cups in cavities since the dried leaves from which the nest is constructed more closely resemble a cup-like open structure. We also recorded the average nest height from the ground, either as the single value reported in the nest descriptions, or, when several values were available, as the mid-height between the lowest and highest nest heights reported for the species, which might correlate with antipredator strategy in different habitats [4,16,41,74–76].

**Table 2.** Taxonomic distribution of nest type and location. (For each family, we note in bold the number of species and genera included in this study, and the number in parentheses indicates the total number of species or genera recognized by the *Handbook of the Birds of the World* and BirdLife International (2022).)

family	species	genera	nest type		branch	hanging	location		
			cup	dome			cavity	bank/rock	ground
Pipridae	**42** (53)	**16** (17)	42	0	42	0	0	0	0
Cotingidae	**48** (67)	**23** (24)	48	0	44	0	0	4	0
Tityridae	**26** (39)	**6** (7)	10	16	9	14	3	0	0
Oxyruncidae	**1** (1)	**1** (1)	1	0	1	0	0	0	0
Onychorhynchidae	**8** (9)	**3** (3)	0	8	0	8	0	0	0
Tyrannidae	**341** (450)	**98** (102)	237	103	188	64	38	18	23
**all**	**466** (619)	**147** (154)	339	127	283	86	41	22	23

#### (iii) Habitat categorization

For each species in the dataset, we followed Tobias *et al*. [51] in assigning one of three habitat types: closed (dense habitats in forest), semi-open (forest edges, dense understory, thickets or shrubland), or open (deserts, grassland, low shrubs, rocky habitats, seashores and cities).

#### (iv) Ecological, life-history and environmental factors associated with nest types

Based on a literature search, we identified ecological, life-history and environmental traits that might correlate with nest type variation (summarized in table 1). To test for possible correlations between nest type (cup or dome) and these traits in Tyrannida, we compiled data on the following for each species in the dataset: nest location (see above), nest height (see above), nest habitat (see above), clutch size [72]; adult body mass and hand-wing index [77]; beak dimensions [78]; territoriality [77]; elevation [72]; latitude, temperature, and precipitation [77]; and range size [78]. For elevation, we recorded the average elevational distribution of the species, as reported in HBW Alive [72], which incorporates information from local field guides. For latitude, we recorded the centroid latitude, which is the geometric centre of the species range (restricted to breeding and resident range) as described by Tobias *et al*. [78]. For temperature and precipitation, we recorded, for each species’ breeding range, the average and annual variation in temperature and precipitation using the WorldClim v.1 database at 10 min resolution for 1970–2000 [79], as reported in Sheard *et al*. [77].

### (b) Phylogenetic comparative methods

We downloaded a 1000-tree subset of Tyrannida topologies from birdtree.org [69], based on the Hackett *et al*. [80] backbone. We then used TreeAnnotator [81] to obtain a maximum clade-credibility tree, forming the species-level phylogeny for our comparative analyses.

#### (i) Nest type evolution and phylogenetic signal

We explored the evolutionary shifts between cup and dome nest types in Tyrannida using the ‘Multistate’ module in the program BayesTraits [82]. As a first step, a maximum-likelihood estimation was run on the binary nest type dataset to obtain approximate transition rate values between cups and domes, according to which we picked prior settings for our Markov chain Monte Carlo (MCMC) run. We then employed an exponential prior with a mean of 10 and ran a chain of 1 010 000 iterations with an initial burn-in of 10 000 runs, and a sampling period of 1000, for a total of 1000 generations. To visualize the ancestral state reconstructions of binary nest type (cup or dome) as well as nest type and location (based on seven combinations: cup/branch, cup/banks or rocks, cup/cavity, cup/ground, dome/branch, dome/ground, dome/hanging), we also performed 100 rounds of stochastic character mapping using the function make.simmap [83] on an all-rates-different model (electronic supplementary material, table S2) in the R package phytools 1.2-0 [84], which uses MCMC simulations. We used iTOL v. 6.7.4 [85] to visualize habitat. We calculated the phylogenetic signal in the presence of a cup or dome nest by using the Fritz & Purvis [86] D estimator for binary traits, applying the function ‘phylo.d’ from the R package *caper* [87]. We ran 1000 simulations to test whether observed values of D were significantly different from those obtained if we assume no phylogenetic structure to the data (*D* = 1) or if evolution of this trait is consistent with Brownian motion (*D* = 0).

#### (ii) Habitat

To quantify the macroevolutionary patterns associated with the three habitat variables (habitat density: closed = 1, semi-open = 2, open = 3), we again ran a BayesTraits ‘Multistate’ model. Model parameters were set to 1 010 000 iterations, with an initial burn-in of 10 000 iterations and a sampling period of 1000, for a total of 1000 iterations, and priors for the transition rates were set to an exponential distribution with a mean of 10.

#### (iii) Evaluating coevolution of nest architecture and habitat type

To investigate a possible coevolutionary association between nest architecture and nesting habitat, we employed the ‘Discrete’ program with MCMC [88] as implemented in BayesTraits. Since the Discrete module only allows traits with binary states to be modelled, we binarized our habitat classification under the following three habitat schemes: (i) closed habitats versus semi-open and open habitats; (ii) closed and semi-open habitats versus open habitats; and (iiii) closed habitats versus open habitats, for which all species in the semi-open habitat class were reassigned to ‘closed’ or ‘open’ based on key terms in the habitat description (i.e. closed = forest, second-growth forest, woodland, dense vegetation; open = light woodland, borders, scattered trees, shady plantations, cultivated areas, pastures) and on discussions with experienced field biologist colleagues. We compared continuous-time Markov models of dependent and independent evolution for nest type against each binarized habitat dataset to explore if nest architecture and habitat are likely to have evolved in association with one another. We set the rate parameter priors as an exponential distribution with a mean of 10 and ran MCMC chains for 200 million iterations, sampling every 200 000th generation, and with a burn-in period of 20 million generations. We used the MCMC Trace Analysis tool (TRACER) v1.6 [89] to review effective sample size (ESS) estimates for posterior probability distributions; all analyses reported ESS > 400. To compute marginal likelihood values, we employed a stepping-stone sampler in MCMC [90], which used 200 stones and ran each stone for 200 000 iterations. We then used these likelihood scores to calculate Bayes factors for our model comparisons.

#### (iv) Testing for potential correlates of nest type

Finally, to evaluate potential ecological, life-history and environmental correlates of nest type (cup or dome), we conducted phylogenetic logistic regressions using the package phylolm [91] in R version 4.0.2. Phylogenetic residuals were modelled under Brownian motion, and the searching space bound was set at 20. To improve interpretability of the model output, all continuous predictor variables were rescaled to have a mean of 0 and a variance of 1 prior to analysis; in addition, clutch size, body mass, and range size were transformed by the natural log, elevation and nest height were square-root transformed, and latitude was considered in absolute value (i.e. distance from equator). Multicollinearity was evaluated using the variance inflation factor (VIF); all VIF values for models without habitat or with habitat as a binary were below 5, and all VIF values were below 8.

We ran three types of models. First, we assessed the relationship between nest type (cup- versus dome-nesting) and potential ecological and life-history correlates (i.e. drivers) of shifts between these traits: nest habitat, nest height, clutch size, adult body mass, flight ability (hand-wing index (HWI)), beak dimensions and territory defence behaviour (summarized in table 1). We consider these ecological and life-history traits to be potential drivers of nest type variation because they might directly (or indirectly) influence aspects of nest site location, nest construction or nest design. As with the coevolutionary models (see above), we evaluated all three possible classifications of the habitat variables (closed versus semi-open and open; closed and semi-open versus open; closed versus open). We also used a version of the model with habitat as a ternary variable (closed versus semi-open versus open).

Second, we modelled correlations between nest type and a suite of environmental traits (i.e. more precise measurements of habitat, as well as proxies for niche occupancy), including elevation, latitude, range size, average range temperature, average range precipitation, and average breeding range variability in temperature and precipitation (both measures of seasonality). We included adult body mass in this model to control for effects body size (and associated life-history traits, like nest size) might have on how a species responds to environmental factors with respect to nest building [18]. Environmental and life-history traits are summarized in table 1. Overall, we consider environmental traits—like a species' elevation or range size—to be potential consequences of nest type variation because of the purported link between shifts in nest architecture and the colonization of new habitats and ecological niches.

Third, as a check against the potential statistical bias of the small number of macroevolutionary transitions within our dataset, we ran separate models including nest type and each of the unique fixed effects listed above and summarized in table 1.

## 3. Results

### (a) Taxonomic and phylogenetic distribution of nest traits

**Figure 2.**
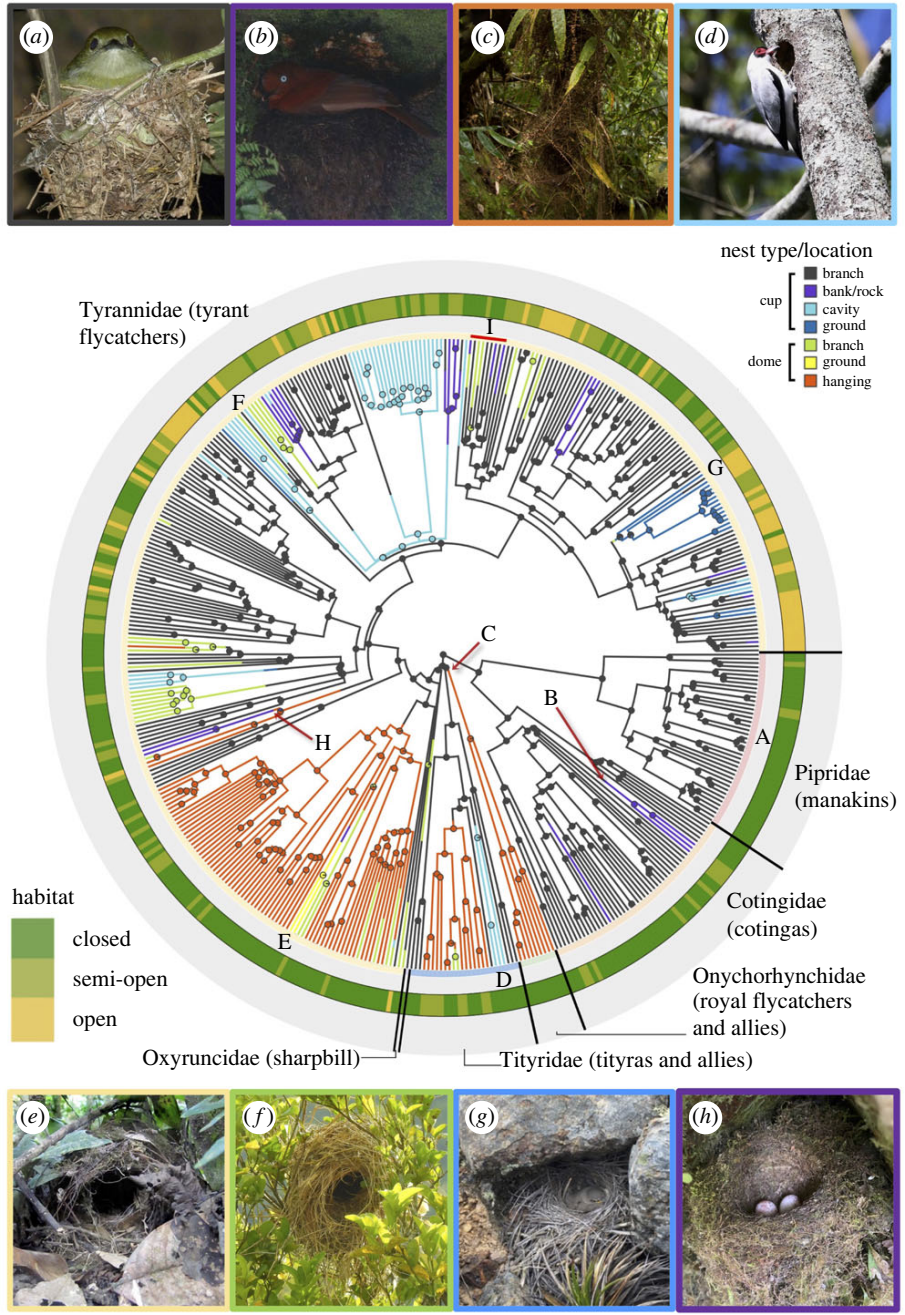
Phylogenetic distribution of nest architecture and location, based on 100 rounds of stochastic character mapping. The external circle represents the habitat type (closed, semi-open, open) across the Tyrannida. Nest type–location ancestral state estimations are depicted on the phylogeny. Examples of nest architecture diversity, in terms of nest type and location, are depicted in the photographs and shown on the phylogeny. (*a*) Cup–branch (white-bearded manakin–*Manacus manacus*); (*b*) cup–banks or rocks (Andean cock-of-the-rock–*Rupicola peruvianus*); (*c*) dome–hanging (Atlantic royal flycatcher–*Onychorhynchus swainsoni*); (*d*) cup–cavity (masked tityra–*Tityra semifasciata*); (*e*) dome-ground (ringed antpipit–*Corythopis torquatus*); (*f*) dome–branch (great kiskadee–*Pitangus sulphuratus*); (*g*) cup–ground (spot-billed ground-tyrant–*Muscisaxicola maculirostris*); and (*h*) transition from dome–hanging to cup–bank (shown in the photo is the cup-building cinnamon flycatcher–*Pyrrhomyias cinnamomeus*). (*i*) A red line highlights members of the chat-tyrant genus *Ochthoeca,* which includes species that build cup and dome nests*.* Photo credits: (*a,b,g,h*) David Ocampo, (*c*) Daniel Perrella, (*d*) John and Milena Beer, (*e*) Gustavo Londoño, (*f*) Juan Felipe León.

Among the studied species (*n* = 466), the cup is the most common nest type (73%; figure 2; table 2), present in all species in the Pipridae (manakins), Cotingidae (cotingas), and Oxyruncidae, most species (70%) in the Tyrannidae (tyrant flycatchers), and some species (38%) in the Tityridae. All species in the Onychorhynchidae build dome nests. Across Tyrannida, most nests are found on branches (61%), though some cotingas nest on banks or rocks, some tityrids nest in cavities or in hanging structures, and tyranids nest in a variety of locations, including in cups and domes on the ground (figure 2; table 2).

The ancestral Tyrannida species probably built a cup nest (*ρ* > 0.999; electronic supplementary material, table S1), in agreement with the ancestral state reconstruction estimates showing that early Tyrannida probably built cup nests located in branches (figure 2). Our analysis of state transition rates indicates that dome nests evolved from cup nests several times across this clade, but that transitions from domes back to cups were less common (table 3; electronic supplementary material, figure S1). Consistent with this result, the stochastic character mapping suggests that the dome nest type independently evolved at least 15 times from cups—within the Tityridae, Onychorhynchidae and Tyrannidae (electronic supplementary material, figure S2)—along with a single transition from dome nests to cup nests, in the clade that includes the cinnamon flycatcher (*Pyrrhomyias cinnamomeus*) (figure 2*h*) and cliff flycatcher (*Hirundinea ferruginea*) (electronic supplementary material, table S2).

**Table 3.** The macroevolutionary transition rates among nest architectural states imply that domes were more likely to evolve from cups, while the reverse was rare. (These values indicate instantaneous transition rates between states and can be interpreted as the relative probability of moving from one state to another. Out of the calculated posterior distribution of 1000 estimated rate values, shown here are the median value, the 2.5th percentile value, and the 97.5th percentile value.)

transition rates		median	2.5th	97.5th
from	to		percentile	percentile
cup	dome	0.0049	0.0029	0.0075
dome	cup	0.0007	0.0000	0.0028

We found support for a strong phylogenetic signal (Fritz & Purvis's D, maximum clade credibility tree: *D* = −0.835, *p*_(D=1)_ < 0.001, *p*_(D=0)_ > 0.999) in nest type, indicating that nest type is more phylogenetically conserved than the Brownian expectation. Nest type is especially highly conserved in manakins (Pipridae) and cotingas (Cotingidae), which build cup nests that are typically placed on branches (figure 2*a*) in closed and semi-open habitats. However, in cotingas, there are two independent origins of nests located in banks or rocks: in the clade comprising the Guianan red-cotinga (*Phoenicircus carnifex*) and the cock–of–the–rocks (*Rupicola rupicola and Rupicola peruvianus*; figure 2*b*), and in the purple-throated cotinga (*Porphyrolaema porphyrolaema*). All of the species in the Onychorhynchidae family build dome nests hanging from branches in closed habitats (figure 2*c*). In Tityridae, species in the genus *Tityra* place their nests in cavities (figure 2*d*), while dome nests hanging from branches are present in the becards (genus *Pachyramphus*), with several species distributed in semi-open habitats. The sharpbill, the single species in the family Oxyruncidae, builds a cup nest placed in branches in closed habitats [92].

The family Tyrannidae contains the most species (73% of species in Tyrannida) and exhibits the greatest nest diversity (figure 2). Ground-nesting species with dome nests are rare: the only examples are two antpipit species (genus: *Corythopis*; figure 2*e*), which are embedded in a clade that typically builds hanging dome nests in closed and semi-open habitats. This clade of hanging dome nesters includes the pygmy tyrants (e.g. genera: *Lophotriccus*, *Hemitriccus)*, tody-flycatchers (e.g. genus: *Todirostrum)*, and flatbills (e.g. genera: *Rhynchocyclus*, *Tolmomyias*). Most of the other transitions from cups to domes occurred in species that build nests on branches (e.g. genera: *Pitangus* and *Myiozetetes*; figure 2*f*). Ground-nesting cup-builders such as ground-tyrants (genus: *Muscisaxicola*) are found primarily in open habitats like deserts, grassland, low shrubs and rocky habitats (figure 2*g*). The tyrant flycatcher family also contains the one estimated transition from domes to cups, in the clade that contains the cinnamon flycatcher (figure 2*h*) and cliff flycatcher; this group is sister to the clade including the dome-nesting orange-banded flycatcher (*Myiophobus lintoni*), ochraceous-breasted flycatcher (*Myiophobus ochraceiventris*), and ornate flycatcher (*Myiotriccus ornatus*). In the Tityridae and Tyrannidae, there are 41 species from 11 genera that build cup nests inside cavities, mostly in closed habitats (table 2).

### (b) No evidence of coevolution between habitat and nest architecture

**Figure 3.**
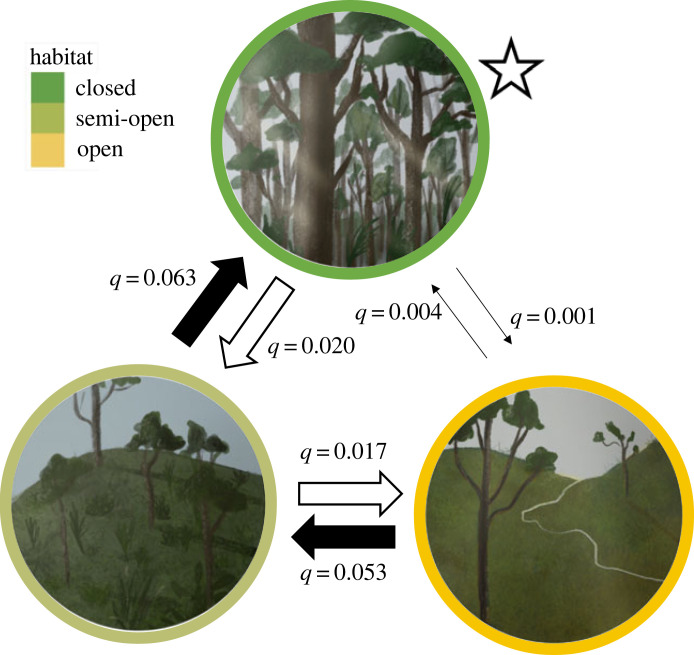
Macroevolutionary transitions among habitat states. The value *q* represents the instantaneous transition rate between states and can be interpreted as the relative probability of moving from one state to another; thin arrows indicate smaller *q* rates (less likely transitions) and thick, black arrows indicate larger *q* rates (more likely transitions). Median rate values are presented here. The star next to the ‘closed’ habitat circle indicates that it is the most probable ancestral state. Original illustrations by Maria Camila León.

An analysis of the macroevolutionary transitions between habitat types yielded no qualitative difference in transition rates between either semi-open and open habitats or semi-open and closed habitats. For this reason, there is no strong rationale for species nesting in semi-open habits to be reassigned to open or closed habitat types, which might be the case if, for example, transitions between semi-open and open habitats were much more common than transitions between semi-open and closed habitats (figure 3). Instead, transitions from open to semi-open habitats, as well as from semi-open to closed habitats, were relatively common (*q* = 0.053 [0.023, 0.099] and *q* = 0.063 [0.043,0.093], respectively, with values representing the distribution median [2.5% percentile, 97.5% percentile]). However, transitions directly between open and closed habitats were much rarer (*q* = 0.001 [0.000,0.003] for closed to open and *q* = 0.004 [0.000,0.020] from open to closed), again underscoring the importance of semi-open habitat as a distinct, biologically meaningful category (electronic supplementary material, table S3).

Within this macroevolutionary transition analysis (figure 3), the predicted Tyrannida ancestral habitat was probably closed (median probability 0.658) or potentially semi-open (median probability 0.284), but it was unlikely to have been open (median probability 0.009).

**Figure 4.**
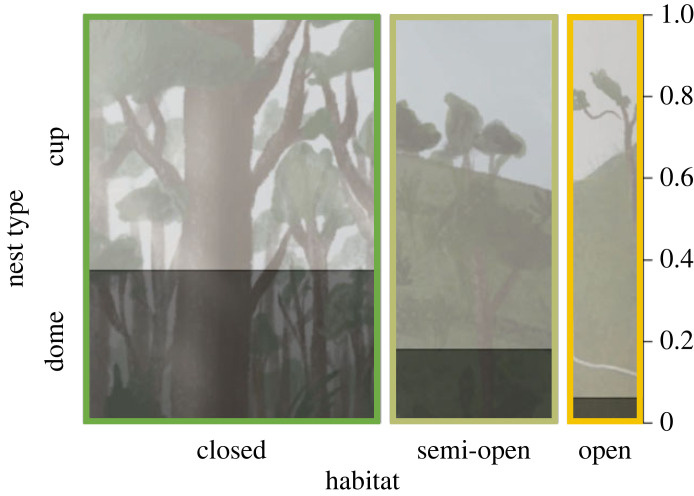
Distribution of cup and dome nests among different habitat types. Original illustrations by Maria Camila León.

Despite an apparent association between habitat and nest type (figure 4)—i.e. domes are proportionally more common in closed than semi-open or open habitats—we found no evidence of coevolution between shifts in nest shape and habitat transitions (electronic supplementary material, table S4). This lack of coevolution holds under different reclassifications of semi-open habitats (electronic supplementary material, table S4).

### (c) Ecological, life-history and environmental correlates of nest type variation

Interspecific variation in nest type within the Tyrannida is apparently unrelated to habitat type, nest height, clutch size, adult body mass, flight ability (HWI), beak dimensions or territoriality, either considered within a single model (electronic supplementary material, tables S5, figure S3) or tested individually (electronic supplementary material, tables S6–S8). Cup and dome nest types are also unrelated to any of the environmental traits assessed here (average range elevation and latitude, average temperature and precipitation, temperature seasonality, precipitation seasonality or range size), both within a single model (electronic supplementary material, table S9, figure S4) or tested individually (electronic supplementary material, table S10).

## 4. Discussion

As the group containing the most speciose family of birds, the tyrant flycatchers and allies offer a compelling clade in which to examine the evolution of nest architecture in passerine birds. Tyrannida species show substantial variation in nest type and nest location (figure 2), with cup and dome nests—in a variety of configurations on the ground, in cavities, on rocks or banks, on branches or hanging—evolving across the phylogenetic tree. Overall, we detected a strong phylogenetic signal in nest type (cup versus dome) (figure 2), suggesting that shifts in nest type are relatively rare, particularly in the cotingas and manakins. The ancestral Tyrannida species probably built a cup nest and lived in forested (i.e. closed) habitat. Across the Tyrannida clade, dome nesting evolved from cup nesting at least 15 times. This is a larger number of nest type shifts than those found in other suboscine passerine lineages, such as the furnariids [5] and antbirds [93], and comparable to the number of shifts in the 71-family Passerida lineage [9]. Within the Tyrannida, we reconstructed only a single reversal from dome nesting back to cup nesting: the cinnamon flycatcher and cliff flycatcher build cup nests, unlike their closest dome-nesting relatives, the orange-banded flycatcher (*Myiophobus lintoni*), ochraceous-breasted flycatcher (*Myiophobus ochraceiventris*) and ornate flycatcher (*Myiotriccus ornatus*). Several Tyrannida lineages ultimately colonized open habitats by initially occupying semi-open habitats (figure 3). Dome nests are often found in closed habitats and seldom in open habitats (figure 4), but this relationship was not statistically significant (electronic supplementary material, table S4): we did not find evidence for coevolution of nest type and habitat. Moreover, we did not detect an association between nest type and any of the ecological, life-history and environmental traits hypothesized to impact nest architecture. Therefore, contrary to our expectations, we found no evidence that shifts in nest type allowed Tyrannida species to colonize new habitats or otherwise expand their ecological niches.

We found no support for coevolution between nest type and habitat in Tyrannida. Why might this be? Even though dome nesters are more common in closed and (to a lesser degree) semi-open habitats than they are in open habitats across the parvorder, cup- and dome-nesting species are found in all three habitat types in the Tyrannidae family [34,35,94–96]. However, within this family, there are relatively few independent shifts between cup- and dome-nesting species, reducing the statistical ability to detect coevolution if it exists. Moreover, in the next two largest families—Cotingidae and Pipridae—all species are cup nesters in closed or semi-open habitats [34]. Thus, one potential explanation for a lack of coevolution between nest type and habitat is that—despite the variation in nest architecture found within this clade—nest type generally shows strong phylogenetic inertia in Tyrannida. It is thus difficult to determine if, when shifts do occur (typically from cup to dome), they are accompanied by a predictable, corresponding shift in habitat type. Another possibility is that our nest and habitat categorizations were too coarse. For example, with respect to nest type, the lighter cup of a white-bearded manakin (*Manacus manacus*; figure 2*a*) differs markedly from the more robust cup of a cinnamon flycatcher (figure 2*h*), despite the birds having similar body masses. Assessing a trait related to nest size [10]—such as nest volume, which tends to be greater in colder climates [18]—may thus be more ecologically and evolutionarily relevant within this context.

We also did not detect a significant relationship between nest type and a suite of ecological, life-history and environmental traits. One possibility is that our analysis overlooked potential important correlates, such as parental care [97], which we excluded because there is no information available for most of the species. For example, in a recent study of nest architecture in more than 3000 passerine species, shifts to cup nesting were associated with decreased investment (i.e. time) in nest building and with increased range sizes and broader thermal niches [7]. Moreover, flight ability and beak dimensions are more likely to be primary drivers of variation in dispersal ability and diet, respectively [77,98], potentially eclipsing any secondary effect they might have on nest construction. In fact, the sharpbill has an elongated and refined beak and builds cups, contrary to our proposed prediction (table 1). While it is certainly plausible that a more detailed analysis could reveal new relationships, the most likely conclusion is that in Tyrannida, phylogenetic history explains a great deal of variation in nest type. Furthermore, one consequence of the strong phylogenetic signal in nest type is that many of our comparative tests also had low statistical power. Thus, an important caveat is that even though we did not detect a significant association between nest type and a variety of relevant ecological, life-history and environmental traits, these variables may nonetheless be predictive on a larger taxonomic scale. Overall, in Tyrannida, many selective forces probably tug at the nest phenotype in varied and unpredictable ways, with no one single factor—including habitat (see above paragraph)—consistently affecting nest type at this taxonomic scale.

The strong phylogenetic signal in nest type, however, does suggest that nest descriptions can be taxonomically informative in Tyrannida. In a recent example of this, within the family Tyrannidae, molecular analysis revealed non-monophyly within *Myiophobus* (a typical cup-nesting genus), and a taxonomic split was proposed, re-assigning three species to a new genus, *Nephelomyias* [99], with closer affinities to another dome-nesting genus, *Myiotriccus*. This taxonomic differentiation was later validated when two of the *Nephelomyias* species were described as building dome nests [100,101]. A similar example involves two former congeners, the dome-nesting great kiskadee (*Pitangus sulphuratus*) and the cup-nesting lesser kiskadee (previously *Pintagus lictor*), which were recently placed in separate genera (as *Pitangus sulphuratus* and *Philohydor lictor*, respectively) based on new molecular phylogenetic studies [102]. Outside Tyrannida, several molecular phylogenies have revealed polyphyly in passerine genera comprising species with divergent nest types, resulting for example in splits in *Ploceus* weaverbirds [103] and *Myrmeciza* antbirds [104]. In this study, we also uncover examples of monophyletic Tyrannida lineages whose closely-related species exhibit shifts in nest type (e.g. figure 2*i*). For example, the chat-tyrant genus *Ochthoeca,* known for typically building cup nests, included species in a recent radiation that shifted to building dome nests [105]. Recent proposals have sought to recognize these dome-nesting species in a separate genus (*Silvicultrix*), supported by molecular data [33]. Shifts in nest type may in fact be a dynamic part of the speciation process in some lineages: in Tyrannida, *Ochthoeca* and *Silvicultrix* would be good candidate taxa for further study.

Across passerines, shifts between cups and domes are common, occurring in parallel in diverse lineages [6,20,93]. We recovered the cup structure as the ancestral nest type for Tyrannida, consistent with an earlier study focusing on Australian lineages of passerines, which included representatives from the Tyriannida clade [9]. In a broader taxonomic context, however, dome nests have been suggested as the ancestral nest type for the entire passerine clade [9]. Therefore, the dome nests we observe in 15 lineages within the families Tityridae and Tyrannidae are perhaps the result of ‘reverse evolution’ in nest type (i.e. these species re-evolved domes from cups). Furthermore, the single transition that we recovered from domes to cups in the lineage leading to the cinnamon flycatcher (figure 2*h*; electronic supplementary material, S2) and cliff flycatcher is intriguing; the scarcity of such shifts makes it statistically difficult to test for associations between these transitions and external factors.

Our ancestral reconstruction of habitats accords with previous findings about the ecological radiation (i.e. expansion of habitat and foraging behaviour) of Tyrannida [35]. Initial divergences between Cotingidae, Pipridae and Tyrannidae and allies occurred in closed habitats during the Oligocene. Then, subsequent radiation events occurred in semi-open and open habitats, promoting large-scale diversification in the following Tyrannidae subclades: Elaeniines, Myiarchines, Tyrannines and Fluvicolines (*sensu* [34]). Within these subfamilies, we observed intriguing patterns in nest architecture evolution (figure 2). These include: high variation in cup location and nest heights (Elaeniines), cups located in cavities (Myiarchines), multiple independent shifts from cups to domes (Tyrannines, Fluvicolinines), cups fully supported in banks (Tyrannines), one species—the piratic flycatcher (*Legatus leucophaius*)—that does not build a nest but instead usurps the dome or pendent nest of various other species (Tyrannines), and novel—in Tyrannida—cup-nesting behaviour on the ground (Fluvicolinae). Although we did not find support in Tyrannida for the hypothesis that shifts in nest architecture explicitly promote expansion into new habitats and ecological niches, nest-building innovation nevertheless seems likely to have contributed to the extraordinary species diversity of other avian families, including Furnariidae [5,20].

Our study suggests that the drivers and consequences of shifts in nest architecture are not straightforward in the clade Tyrannida, perhaps owing in part to the low transition rates between the principal nest architectural types. However, the nest is a complex phenotype that can be influenced by many factors at a microevolutionary scale, and it is possible that the macroevolutionary story might be similarly complex and nuanced in other avian groups. In the future, obtaining more detailed information on behavioural and ecological traits associated with nesting (e.g. competition for nest sites, brood parasitism, chick developmental period) and finer-scale information on environmental conditions at the nest could perhaps elucidate the mechanisms by which avian species evolve new nest designs—and sometimes occupy novel ecological niches. However, there remain vast gaps in our knowledge of the breeding biology of many bird species [106–108]. In Tyrannida alone, the nests for over 100 species have yet to be found or described. This especially highlights the critical importance of detailed field-based studies, rooted in natural history and often carried out on small taxonomic groups in remote regions—particularly in the Neotropics [109], for future work on the evolution of nest design in birds. Finally, our study adds to the growing body of work exploring the myriad influences on nest architecture not just in birds [11,14,26,110] but also in non-avian reptiles [111], amphibians [112], fishes [43,113], mammals [114,115] and insects [116]. Across these taxonomic groups, determining the effects of predation [117], habitat [118], thermal properties [119] and parental care [120] on aspects of nest design is a timely goal.

## Data Availability

The datasets supporting this article is available from: https://doi.org/10.6084/m9.figshare.21980333.v1. Range information data is publicly available from www.birdlife.org; climate data from www.worldclim.org; phylogenetic data from www.birdtree.org. The data are provided in the electronic supplementary material [121].
